# Development and validation of a predictive model in diagnosis and prognosis of primary glioblastoma patients based on Homeobox A family

**DOI:** 10.1007/s12672-023-00726-y

**Published:** 2023-06-23

**Authors:** Zong-Qing Zheng, Gui-Qiang Yuan, Guo-Guo Zhang, Qian-Qian Nie, Zhong Wang

**Affiliations:** 1 Department of Neurosurgery & Brain and Nerve Research Laboratory, The First Affiliated Hospital of Soochow University, 188 Shizi Street, Suzhou, 215006 Jiangsu Province China; 2 Beijing Neurosurgical Institute & Department of Neurosurgery, Beijing Tiantan Hospital Affiliated to Capital Medical University, Capital Medical University, Beijing, China

**Keywords:** Glioblastoma (GBM), Homeobox-A (HOXA), Nomogram, Prognosis, Diagnosis

## Abstract

**Background:**

Homeobox A (HOXA) family is involved in the development of malignancies as either tumor suppressors or oncogenes. However, their roles in glioblastoma (GBM) and clinical significance have not been fully elucidated.

**Methods:**

HOXA mutation and expressions in pan-cancers were investigated using GSCA and Oncomine, which in GBM were validated by cBioPortal, Chinese Glioma Genome Atlas (CGGA), and The Cancer Genome Atlas (TCGA) datasets. Kaplan–Meier analyses were conducted to determine prognostic values of HOXAs at genetic and mRNA levels. Diagnostic roles of HOXAs in tumor classification were explored by GlioVis and R software. Independent prognostic HOXAs were identified using Cox survival analyses, the least absolute shrinkage and selection operator (LASSO) regression, quantitative real-time PCR, and immunohistochemical staining. A HOXAs-based nomogram survival prediction model was developed and evaluated using Kaplan–Meier analysis, time-dependent Area Under Curve, calibration plots, and Decision Curve Analysis in training and validation cohorts.

**Results:**

HOXAs were highly mutated and overexpressed in pan-cancers, especially in CGGA and TCGA GBM datasets. Genetic alteration and mRNA expression of HOXAs were both found to be prognostic. Specific HOXAs could distinguish IDH mutation (HOXA1-7, HOXA9, HOXA13) and molecular GBM subtypes (HOXA1-2, HOXA9-11, HOXA13). HOXA1/2/3/10 were confirmed to be independent prognostic members, with high expressions validated in clinical GBM tissues. The HOXAs-based nomogram model exhibited good prediction performance and net benefits for patients in training and validation cohorts.

**Conclusion:**

HOXA family has diagnostic values, and the HOXAs-based nomogram model is effective in survival prediction, providing a novel approach to support the treatment of GBM patients.

**Supplementary Information:**

The online version contains supplementary material available at 10.1007/s12672-023-00726-y.

## Introduction

Glioblastoma (GBM) is known to be the most malignant brain tumor globally, with a median survival time of only about 14.6 months [1]. Despite undergoing surgical tumor resection, radiotherapy, and chemotherapy, only 9.8% of patients survive after 5 years [2]. The dismal prognosis of GBM is linked to its characteristics, including cell proliferation, invasion, metastasis, and drug resistance. Recent studies have shown that epigenetic regulation plays a crucial role in tumor neoplasia and progression in GBM [3], with IDH mutation and MGMT methylation being significant factors. Thus, investigating further molecular mechanisms and specific biomarkers is crucial to provide better diagnosis and treatment strategies for patients.

Homeobox A (HOXA) genes, which code for transcription factors, are closely related to epigenetic regulation, cell proliferation, and differentiation [4]. HOXA family is distributed in the cluster at chromosome (7p15.3), with HOXA1-7, 9–11, and 13 numbered in sequence along the chromosome 3′ to 5′. During cell development and differentiation, each HOXA family is sequentially activated or silenced from chromosome 3′ to 5′, and the HOXA proteins differently recognize and regulate the transcription of target genes via the homeodomain, which can influence tumorigenesis [5]. The aberrant expression of HOXA family has been confirmed in many malignancies, such as gastric cancer [6], colon cancer [7], lung cancer [8], cervical cancer [9], and hepatocellular carcinomas [10, 11]. In GBM, HOXA1 [12, 13], HOXA2 [13], HOXA3 [13, 14], HOXA5 [15], HOXA6 [14, 16], HOXA7 [16], HOXA9 [16], HOXA10 [17, 18], HOXA11 [19] and HOXA13 [16, 20] were significantly up-regulated, compared to normal tissues. However, only partial HOXA genes exhibited prognostic values in previous studies.

HOXA1 exhibited predictive value in the Kaplan–Meier analysis of a single GBM database [21]. HOXA10 was also validated in the Kaplan–Meier analysis but excluded in further multivariate Cox survival analysis [22]. Besides, the methylation of HOXA3 contributed to a longer survival time in high-grade glioma patients [23]. The combination of HOXA9 and HOXA10 expression was also associated with a shorter overall survival time (OS) in 78 pediatric patients with GBM [24]. Besides, antisense RNA of HOXAs also participated in the progression of GBM [25–27], and the prognostic values of these antisense RNAs were also explored [28, 29]. Overall, the above-reported studies mainly focused on a single database, and the roles of the whole HOXA family in GBM were still unclear. Thus, The functions and potential clinical values of HOXA family in GBM require more comprehensive exploration.

With the development of sequencing technologies and the accumulation of much clinical data, this study aims to disclose the values of HOXAs in GBM further. First, we evaluated the mutation and expression of HOXAs in pan-cancers. Then we validated the mRNA expression difference in multiple GBM databases, including the Chinese Glioma Genome Atlas (CGGA) and The Cancer Genome Atlas (TCGA). The prognostic values of HOXA family at genetic alteration and mRNA expression were explored using Kaplan–Meier analysis. The correlation between HOXA family and important clinical factors, including IDH mutation and molecular GBM subtypes, was first investigated in our research. Then multivariate COX survival analysis in CGGA and TCGA databases screened out the independent prognostic HOXA genes, which were further validated by the least absolute shrinkage and selection operator (LASSO) regression, quantitative real-time PCR (qRT-PCR), and immunohistochemical (IHC immunochemistry) staining. Finally, an HOXAs-based nomogram survival prediction model was constructed and evaluated in both training and validation cohorts. It is hoped that the exploration of HOXA family will provide new approaches for diagnosing and treating GBM patients.

## Materials and methods

### Ethics statement

Human GBM and adjacent normal tissues were provided by The First Affiliated Hospital of Soochow University, with the approval of the Institutional Review Board (IRB) at The First Affiliated Hospital of Soochow University. Written informed consent was obtained from all patients in line with the World Medical Association's Declaration of Helsinki. The study design and following experiments were under the guidance of the IRB of the First Affiliated Hospital of Soochow University.

### Data download and processing

**Fig. 1:**
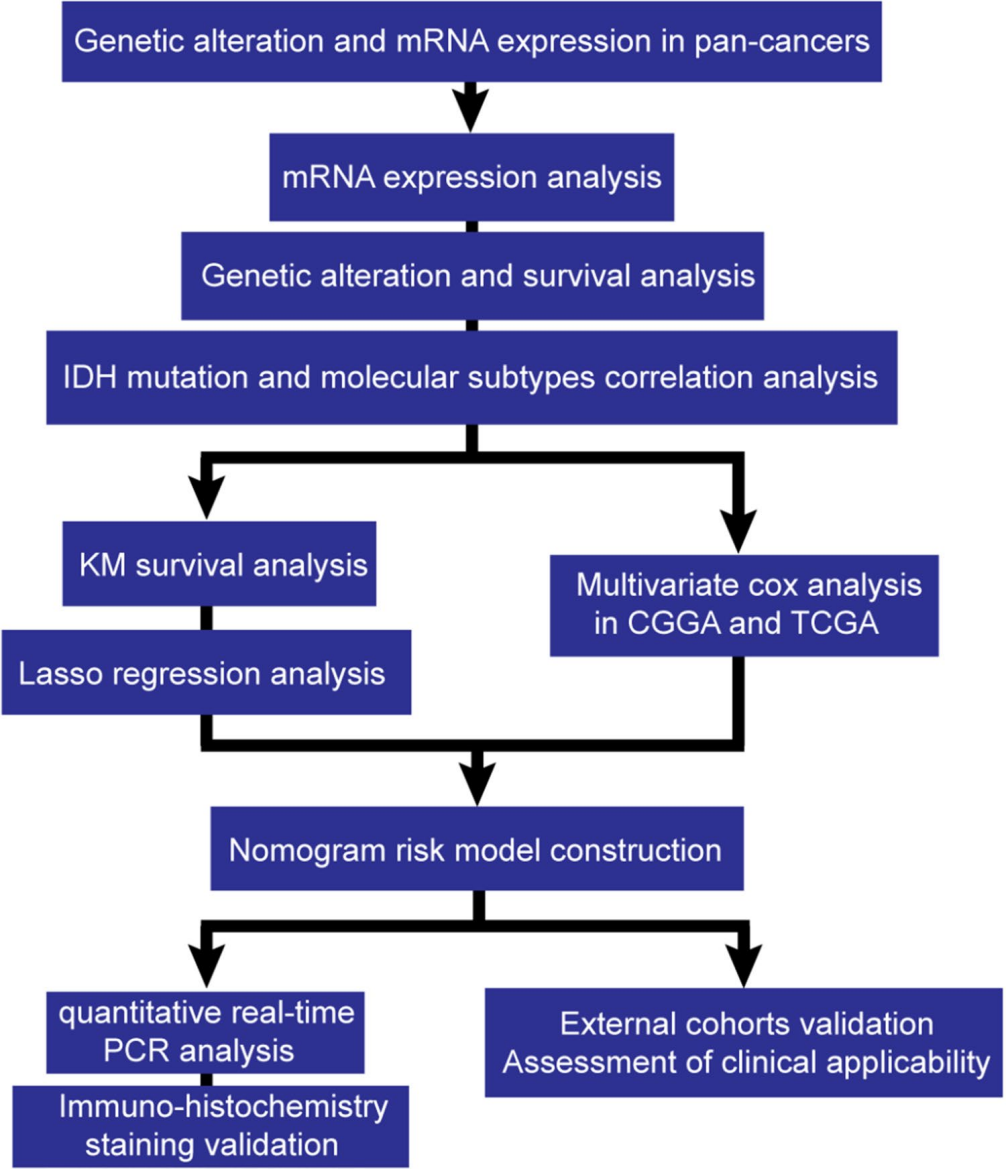
Flow diagram of the study

The study flow diagram is presented in Fig. 1. TCGA provided the mRNA expression of HOXAs and related survival time in pan-cancers. Then we utilized R and packages to analyze the aberrant expression and prognostic values of HOXAs in pan-cancers. While investigating HOXAs in GBM, CGGA database provided over 2,000 samples from Chinese cohorts and 1018 mRNA sequencings with clinical data. We download the whole data, including "mRNAseq_693" [30, 31] and "mRNAseq_325" [32, 33], from the website. Then we screened out the WHO IV primary GBM data, which had 216 samples with HOXAs family expression and clinical information (Supplementary Table 1, 4). The "mRNAseq_693" database has not detected the expression of HOXA6, so we just used the "mRNAseq_325" database to analyze HOXA6, which was 83 GBM samples (Supplementary Table 5). Clinical data included gender, age, OS, censor, radio/chemotherapy, and IDH mutation. The validation dataset was downloaded from the TCGA database. Recurrent GBM was excluded, and 262 patients with primary GBM were included for validation. TCGA clinical data included gender, age, OS, censor, and IDH mutation (Supplementary Table 2, 6), corresponding to the CGGA. All the above information is exhibited in Supplementary Table 1, 2. Comprehensive analyses, including Kaplan–Meier, ROC, COX survival analyses, and proportional hazards assumption tests, were conducted by R software on these HOXA members. The specific R packages used are shown in Supplementary Table 3. We used Student’s *t*-test in two groups comparison and one-way analysis of variance in three or more groups. *P* < 0.05 was a statistically significant difference.

### Oncomine

Oncomine [34], a public database of cancer microarray information analysis, was used to study various HOXAs mRNA expressions in different cancers. The thresholds were set before we analyzed the HOXAs family in GBM. The thresholds were set as p-value < 1e−4, fold change = all, and gene rank in the top 5%. The statistical procedure used Student’s *t*-test.

### Gene Set Cancer Analysis (GSCA) database

GSCA is a database integrating over 10,000 multidimensional genomic data across 33 cancer types from TCGA to search, investigate and explore the gene set cancer analysis related to mRNA expression, mutation, immune infiltration, and drug resistance [35]. We performed Gene set level analysis of HOXAs in GBM to explore the degree of genetic alteration and cancer pathway activity. *P* < 0.05 was considered statistically significant.

### cBioPortal

cBioPortal is a huge gene expression and alteration analysis website based on TCGA databases [36]. We analyzed the data of Glioblastoma Multiforme (TCGA, Firehose Legacy) to explore the HOXAs family gene mutation and used the survival analysis to figure out the relation between mutation and prognosis. The mRNA expression z-scores relative to diploid samples were set at 1.5. The p-significant value was lower than 0.05. The statistical procedure used Student's t-test.

### GlioVis

GlioVis was a website based on the TCGA and CGGA databases, including the molecular GBM subtypes data [37]. We analyzed the different HOXA expressions and molecular GBM subtypes in the TCGA-GBM database. The results and raw data expressions were also downloaded. We used Tukey's Honest analysis in three or more groups. *P* < 0.05 was a statistically significant difference.

### Clinical tissue samples

Primary GBM samples and normal brain tissues were provided by the department of neurosurgery of the First Affiliated Hospital of Soochow University. The radiologists confirmed the neuroimaging features of these primary GBM patients. The neurosurgeons further diagnosed and resected the GBM tissues. Pathologists provided a further histological examination of these samples. Under the guidance of IRB at The First Affiliated Hospital of Soochow University, the clinical samples were preserved in liquid nitrogen for further investigation.

### RNA extraction and qRT-PCR

Total RNA from samples was extracted by TRIzol reagent (Biosharp, China) and evaluated on a NanoDrop One spectrophotometer (Thermo Fisher Scientific, USA). 1 μg of total RNA was used as the template for the first strand cDNA synthesis using the transcriptor first strand cDNA synthesis kit (F0202, Lablead, China). qRT-PCR was performed with a Green Fast ROX II Mixture kit (A304-01, GenStar, China) on Applied Biosystems QuantStudio 5 (Thermo Fisher Scientific, United States). The groups were normalized based on GAPDH and the primer sequences used were listed: HOXA1, 5ʹ‐CGGCTTCCTGTGCTAAGTCT‐3ʹ (F) and 5ʹ‐TTCATTGTGCCATCCATCAC ‐3ʹ (R); HOXA2, 5ʹ‐GCGCCTGAGAACTGCTTACA‐3ʹ (F) and 5ʹ‐TGTGCTTCATCCTCCGGTTC ‐3ʹ (R); HOXA3, 5ʹ‐TCATTTAAGAGCGCCTGGACA‐3ʹ (F) and 5ʹ‐GAGCTGTCGTAGTAGGTCGC ‐3ʹ (R); HOXA10, 5ʹ‐TCACGGCAAAGAGTGGTC‐3ʹ (F) and 5ʹ‐AGTTTCATCCTGCGGTTCTG ‐3ʹ (R); GAPDH, 5ʹ‐TGACTTCAACAGCGACACCCA‐3ʹ (F) and 5ʹ‐CACCCTGTTGCTGTAGCCAAA ‐3ʹ (R). The expressions were calculated using the −ΔCT method.

### Immunohistochemistry staining

The sections were dewaxed, antigen-retrieved, and blocked. Then, specific antibodies (anti-HOXAs: HOXA1: ab230513; HOXA2: ab229960; HOXA3: ab230879; HOXA10: ab191470, Abcam) covered the sections at 4 °C overnight. After washing three times with PBS, specific secondary antibodies covered the sections for 1 h at 37 °C with horseradish peroxidase, then immersed in diaminobenzidine (DAB) and counterstained with hematoxylin for 2 min. A microscope (BX50/BX-FLA/DP70, OLYMPUS) was used to observe the staining signals. ImageJ Pro (Media Cybernetics, USA) was used by a technician (blinded to the experimental groupings) to quantify the integrated optical densities.

### Statistical methods

The univariate Cox analysis screened out significant clinical factors and HOXAs expressions with a p-value threshold of 0.05. Then the multivariate Cox analysis further explored the association between survival and previous significant factors. *P* < 0.05 was considered statistically significant.

## Results

### High mutation and expression of HOXAs in pan-cancers

**Fig. 2:**
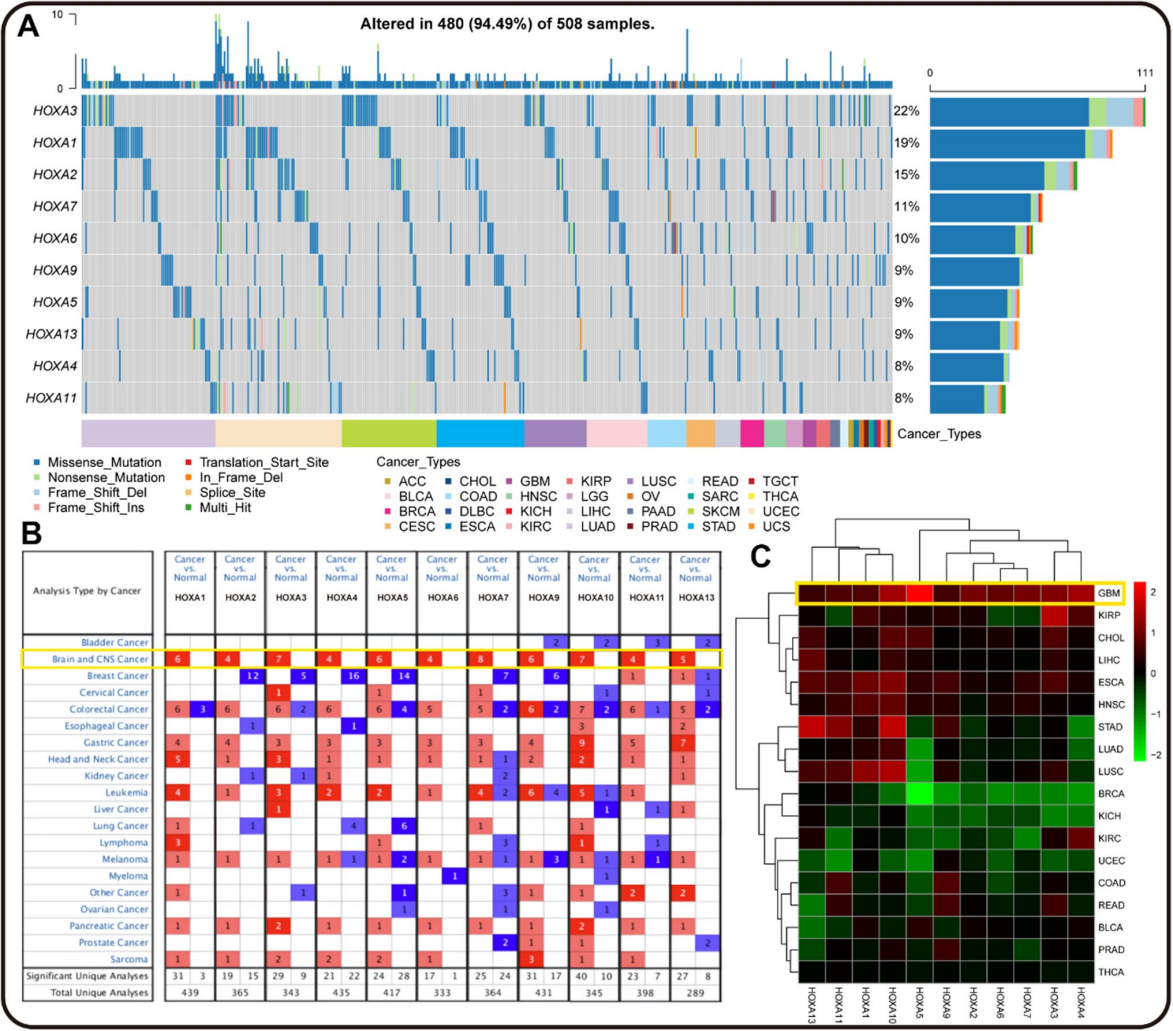
Genetic alteration and mRNA expression of HOXA family in pan-cancers. **A** The Heterozygous mutations of HOXAs in pan-cancers by GSCA. The total altered ratio was up to 94.49%. **B** The numbers of Oncomine datasets were exhibited with mRNA over-expression (red) or under-expression (blue) of HOXAs compared with normal tissues by Oncomine. The Brain and CNS cancers group was significantly overexpressed, highlighted by the yellow box. **C** Visualized heatmap of HOXAs in TCGA pan-cancers data by "pheatmap" R package (over-expression (red) and under-expression (green)). The most expression of HOXAs was in the GBM group, marked with the yellow box

To investigate the roles of the HOXA family in various malignancies, we first used GSCA to perform a genetic alteration analysis based on their single nucleotide variants. As illustrated in Fig. 2A, the top 10 mutated HOXA genes occupied 94.49% of 508 samples, and GBM was the fifth largest mutant group. Furthermore, we also visualized the mRNA expression of HOXA family in pan-cancers and normal tissues by Oncomine and TCGA databases by R software. And we found that compared to normal samples, all HOXA genes were significantly up-regulated in Brain and CNS cancers (Fig. 2B). A similar result was also indicated in GBM than in other cancers (Fig. 1C). As shown in Table 1, the over-expression of HOXA1 in GBM was over 2.292 fold-change. Meanwhile, at least two different databases confirmed 2.4 more fold-change of HOXA3, HOXA5, HOXA9, and HOXA10 in GBM. The fold-change of HOXA5 and HOXA13 was up to 6.686 and 4.481in GBM. These findings suggested that the HOXA family might serve critical roles in pan-cancers, particularly in GBM.

**Table 1:** Differential mRNA expression of HOXAs in GBM, compared to normal tissues in Oncomine

	Samples	Fold change	*P* value	*t*-test	Refs.
HOXA1	Glioblastoma	4.465	4.04E−14	8.677	Sun brain [38]
	Glioblastoma	2.292	4.67E−22	20.961	TCGA brain [39]
HOXA2	Glioblastoma	1.325	7.13E−164	38.716	TCGA brain 2 [39]
HOXA3	Glioblastoma	6.500	5.84E−8	7.596	Lee brain [40]
	Glioblastoma	6.505	1.79E−15	10.291	Sun brain [38]
	Glioblastoma	2.495	2.22E−8	8.756	Murat brain [41]
HOXA4	Glioblastoma	1.325	5.07E−164	38.747	TCGA brain 2 [39]
HOXA5	Glioblastoma	4.270	2.04E−9	13.166	TCGA brain [39]
	Glioblastoma	2.752	9.21E−8	9.273	Murat brain [41]
HOXA6	Glioblastoma	1.325	4.57E−164	38.756	TCGA brain 2 [39]
HOXA7	Glioblastoma	6.686	1.89E−16	10.478	Sun brain [38]
HOXA9	Glioblastoma	6.688	1.05E−5	5.676	Lee brain [40]
	Glioblastoma	2.525	9.98E−10	6.929	Murat brain [41]
HOXA10	Glioblastoma	15.816	8.35E−7	6.523	Lee brain [40]
	Glioblastoma	2.996	2.68E−9	9.284	Murat brain [41]
	Glioblastoma	2.467	1.47E−12	13.987	TCGA brain [39]
HOXA11	Glioblastoma	1.325	3.13E−164	38.791	TCGA brain 2 [39]
HOXA13	Glioblastoma	4.581	7.94E−5	4.574	Lee brain [40]

### High expression of HOXAs at mRNA level in TCGA and CGGA GBM databases

**Fig. 3:**
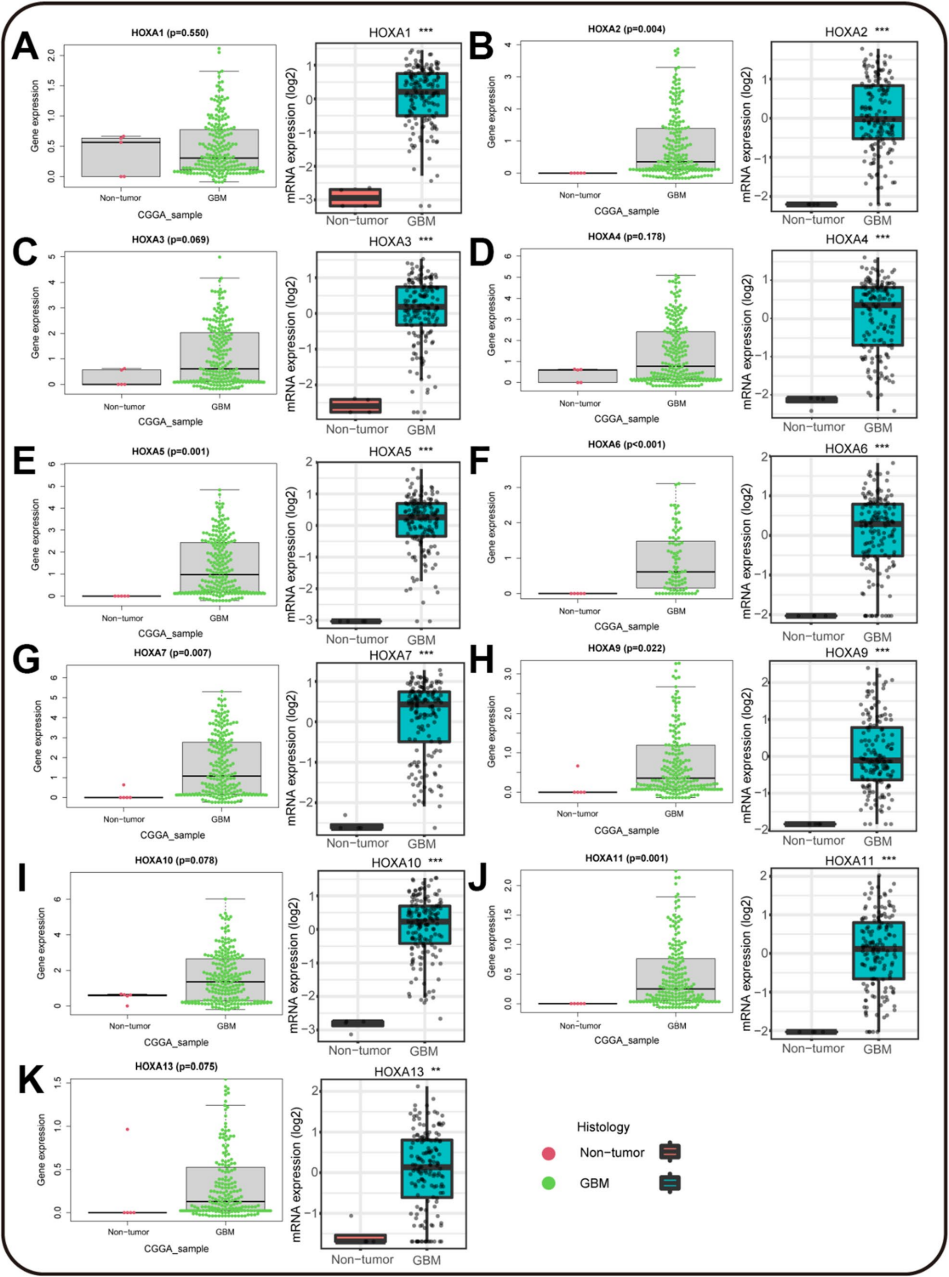
Over-expression of HOXA family in CGGA and TCGA GBM databases. **A**–**K** The mRNA expression of HOXA family in CGGA database was analyzed in the "ggpubr" R package. The red dots represented the normal samples, and the green dots represented the GBM samples. The mRNA expression analysis in TCGA database was provided by GlioVis. Normal samples were in the red bar, and GBM samples were shown in the blue bar. (*P < 0.05, **P < 0.01, ***P < 0.001)

In terms of brain malignancies, GBM is the most dangerous tumor. We further used two different databases to confirm the expression pattern of HOXA family in GBM. CGGA provided the mRNA sequencing of 209 GBM patients, and we used the "beeswarm" R package to conduct the analysis. We found that all HOXA members were significantly up-regulated in GBM samples of CGGA. Besides, we also analyzed the mRNA expression of HOXAs by GlioVis, including 538 GBM patients from TCGA. Compared to normal samples, all HOXA members also presented a higher expression in GBM (Fig. 3A–K). Overall, the up-regulation of HOXAs at mRNA level in GBM was double validated and suggested that HOXA family was worth further exploration in the diagnosis and treatment of GBM patients.

### Prognostic genetic mutation and mRNA expression of HOXA family in GBM

**Fig. 4:**
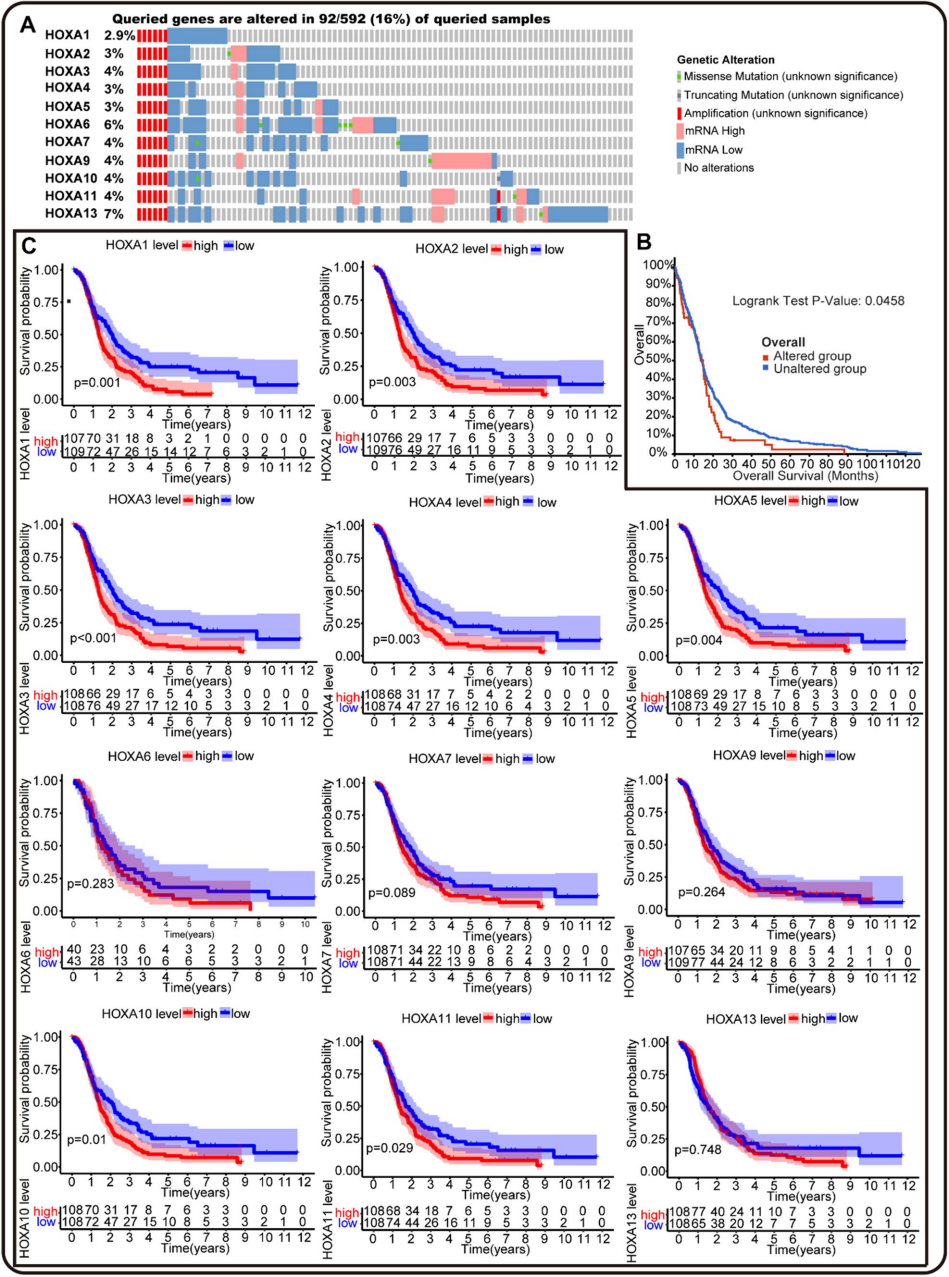
The prognostic values of HOXAs genetic alteration and mRNA expression in GBM. **A** Aberrant genetic mutations of HOXAs in GBM by cBioPortal. The mutation of HOXAs members in GBM samples was 16% (95/592). **B** Prognostic value of HOXAs genetic alterations in overall survival time of GBM patients (p = 0.0458), with 87 altered samples and 493 unaltered samples included. **C** The Kaplan–Meier analysis of HOXAs mRNA over-expression (red) or under-expression (blue) in overall survival time of GBM patients by "survival", "survminer" R packages. (*P < 0.05, **P < 0.01, ***P < 0.001)

To further determine the prognostic value of HOXA family in GBM, we analyzed the mutation and its correlation with the survival time of GBM patients in Glioblastoma Multiforme (TCGA, Pan-Cancer Atlas) data via cBioPortal. As shown in Fig. 4A, the mutation rate of HOXA family was 16% (92/592), ranging from 2.9% to 7% in GBM. And the mutation of HOXA family was significantly correlated to the worse overall survival time of GBM patients (Fig. 4B). These results suggested the genetic alteration of HOXA family may play a critical role in the progression of GBM.

Moreover, we also conducted a Kaplan–Meier survival analysis of HOXA family in the GBM datasheet from CGGA. In Fig. 4C, the plots revealed that the over-expression of HOXA1-5, HOXA10, and HOXA11 contributed to the shorter survival time of GBM patients, while the mRNA expression of HOXA6, HOXA7, HOXA9, and HOXA13 showed no correlation. The above survival analyses at genetic and mRNA levels suggested that HOXA family might act as oncogenes in GBM.

### HOXA family was correlated with IDH mutation and molecular subtypes in GBM

**Fig. 5:**
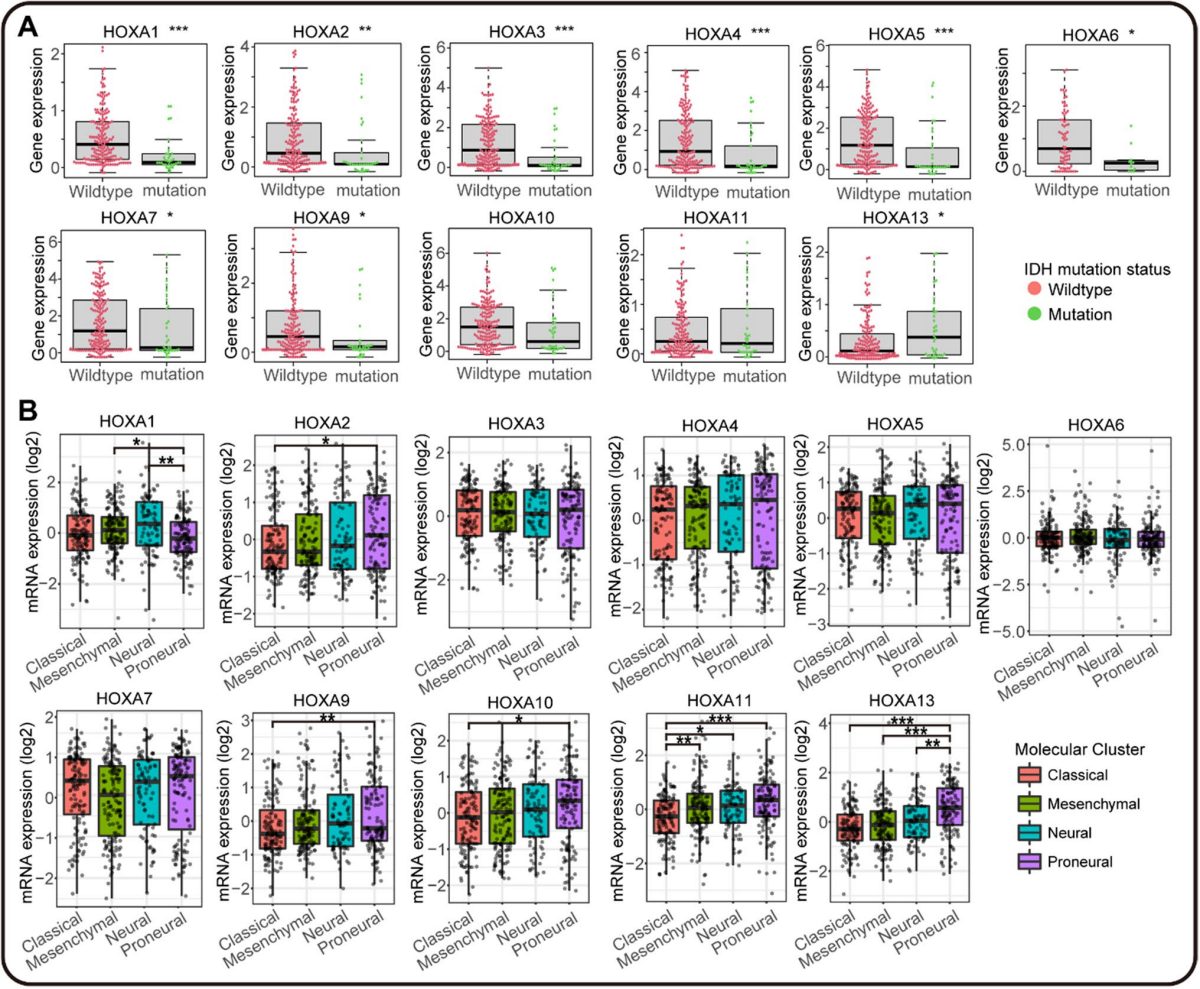
IDH mutation and molecular GBM subtypes correlation analyses in HOXA family **A** IDH mutation in mRNA over-expression or under-expression of HOXAs from CGGA by "beeswarm" R package. The red dots represented the normal samples, and the green dots represented the GBM samples. **B** The molecular GBM subtypes distinction analysis in different HOXAs expression by GlioVis. The molecular cluster of GBM included Classical, Mesenchymal, Neural, and Proneural subtypes. (*P < 0.05, **P < 0.01, ***P < 0.001)

IDH mutation was widely considered a standard clinical indicator of glioma progression and prognosis [42]. HOXA family was distributed in the cluster at chromosome (7p15.3), which correlated with IDH-wildtype classification. Meanwhile, our findings also confirmed the prognostic mutation and expression of HOXA family in GBM. Hence, we further explored the potential relationship between IDH mutation and HOXA family. As shown in Fig. 5A, higher expression of HOXA1, HOXA3, HOXA4, HOXA5 (*P* < 0.0001), HOXA2 (*P* < 0.001), HOXA6, HOXA7, HOXA9 (*P* < 0.05) and lower expression of HOXA13 (*P* < 0.05) were closely related to IDH wildtype. And HOXA10 and HOXA11 showed no difference in IDH mutation and wild-type. These data indicated that most HOXA genes had relevance to IDH mutation, which might contribute to a more precise diagnosis and treatment of GBM patients.

Molecular subtypes were another advancement in the clinical treatment of GBM. There were four different types of GBM classified by specific pathological features, including Classical, Mesenchymal, Neural, and Proneural subtypes [43]. Hence, we also analyzed the correlation between HOXA family and molecular GBM subtypes by GlioVis. As depicted in Fig. 5B, we found that HOXA1 had significantly differential expression in GBM between Proneural subtype and Mesenchymal, Neural subtypes. HOXA2, HOXA9, and HOXA10 all showed a distinct difference between Proneural and Classical subtypes of GBM. Meanwhile, HOXA11 showed a substantial difference between Classical subtype and Mesenchymal, Neural, Proneural subtypes. Besides, there was also a differential expression of HOXA10 between Proneural subtype and Classical, Neural, Proneural subtypes. Together, these results indicated that HOXA family was closely related to molecular GBM subtypes, which may contribute to the classification and treatment of GBM.

### HOXA1/2/3/10 were independently associated with the survival time of GBM patients in CGGA and TCGA

**Fig. 6:**
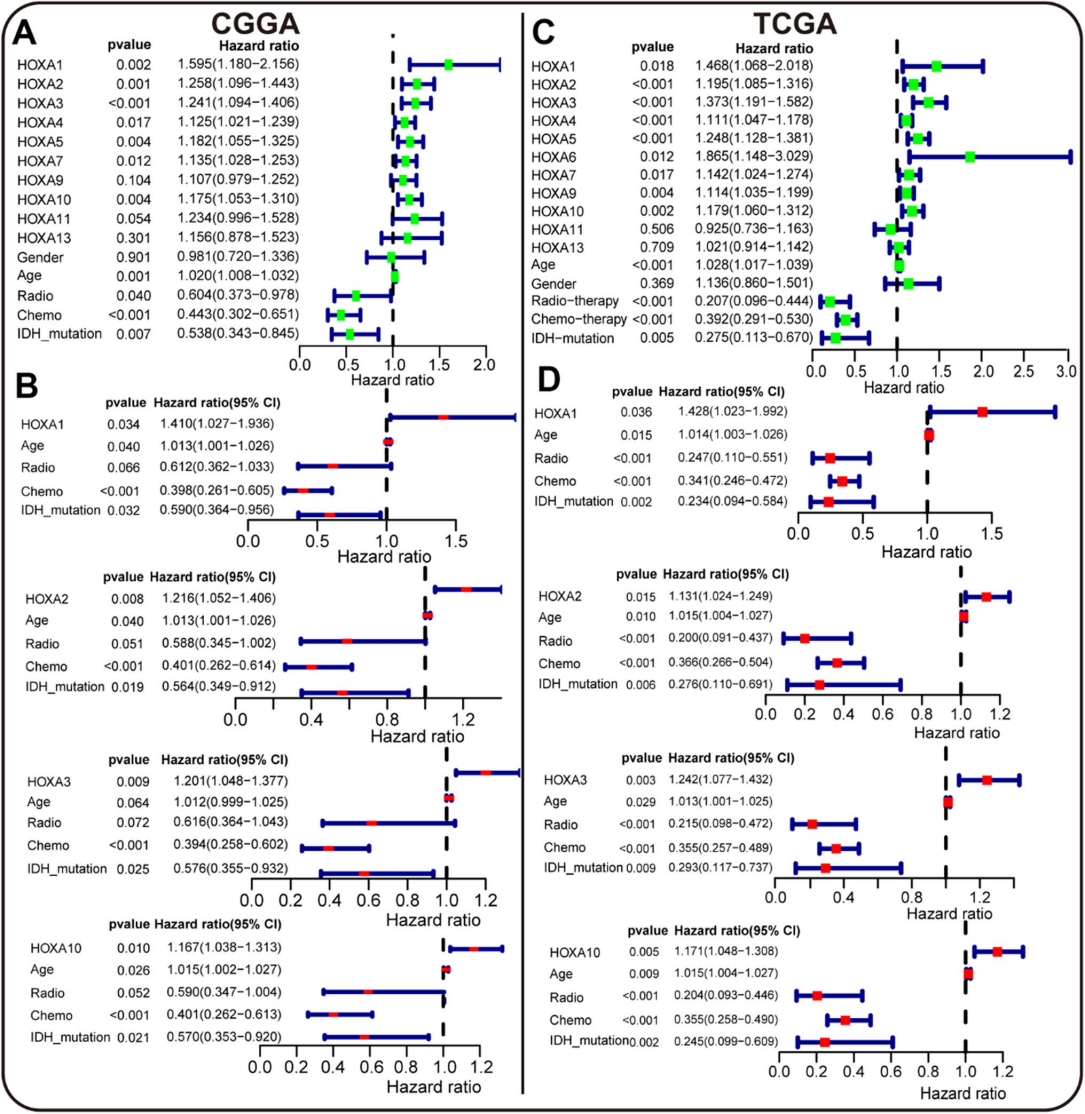
Independent prognostic values of HOXA family in CGGA and TCGA databases**. A**, **C** The univariate survival analysis of HOXAs and clinical factors in CGGA and TCGA databases by "survival", and "survminer" R packages. HOXA1-7, HOXA9-11, HOXA13, gender, age, radiotherapy, chemotherapy, and IDH mutation were included. (The dots in the plots were green). **B**, **D** Further multivariate survival analysis of prognostic HOXAs and clinical factors in CGGA and TCGA databases. HOXA1-3, HOXA10 in CGGA, HOXA1-7, HOXA10 in TCGA, gender, age, radiotherapy, chemotherapy, and IDH mutation were included. (The dots in the plots were red.)

To further explore the prognostic values of HOXA family in GBM, we conducted a comprehensive survival analysis in multi-databases (CGGA and TCGA) using the "survival" and "survminer" R packages. We performed the Cox survival analysis, including HOXA family, gender, age, radiotherapy, chemotherapy, and IDH mutation. First, in CGGA database, the univariate analysis revealed that HOXA1-7, HOXA10, age, radiotherapy, chemotherapy, and IDH mutation played a prognostic role in GBM (Fig. 5a). We analyzed HOXA6 in the "mRNAseq_325" CGGA datasheet, and HOXA6 may not correlate with the survival time of GBM patients (Supplementary Fig. 1A, B). Then prognostic HOXAs, age, radiotherapy, chemotherapy, and IDH mutation were included to perform the further multivariate survival analysis. The results indicated that HOXA1-3 and HOXA10 had independent prognostic values in GBM (Fig. 6B). Similarly, in TCGA, we found that HOXA1-10, age, radiotherapy, chemotherapy, and IDH mutation were related to the survival time of GBM patients (Fig. 6C). Further multivariate analysis confirmed the independent prognostic roles of HOXA1-6, HOXA9, and HOXA10 in GBM (Fig. 6D, Supplementary Fig. 1C–F). By merging the results of the two databases, we confirmed that HOXA1, HOXA2, HOXA3, and HOXA10 were the final double-validated independent survival indicators. Overall, in line with previous results of transcriptomics and Kaplan–Meier survival analysis, HOXA1/2/3/10 were the novel predictive factors in the diagnosis and treatment of GBM.

### Construction of Nomogram survival prediction model based on prognostic HOXAs in GBM

**Fig. 7:**
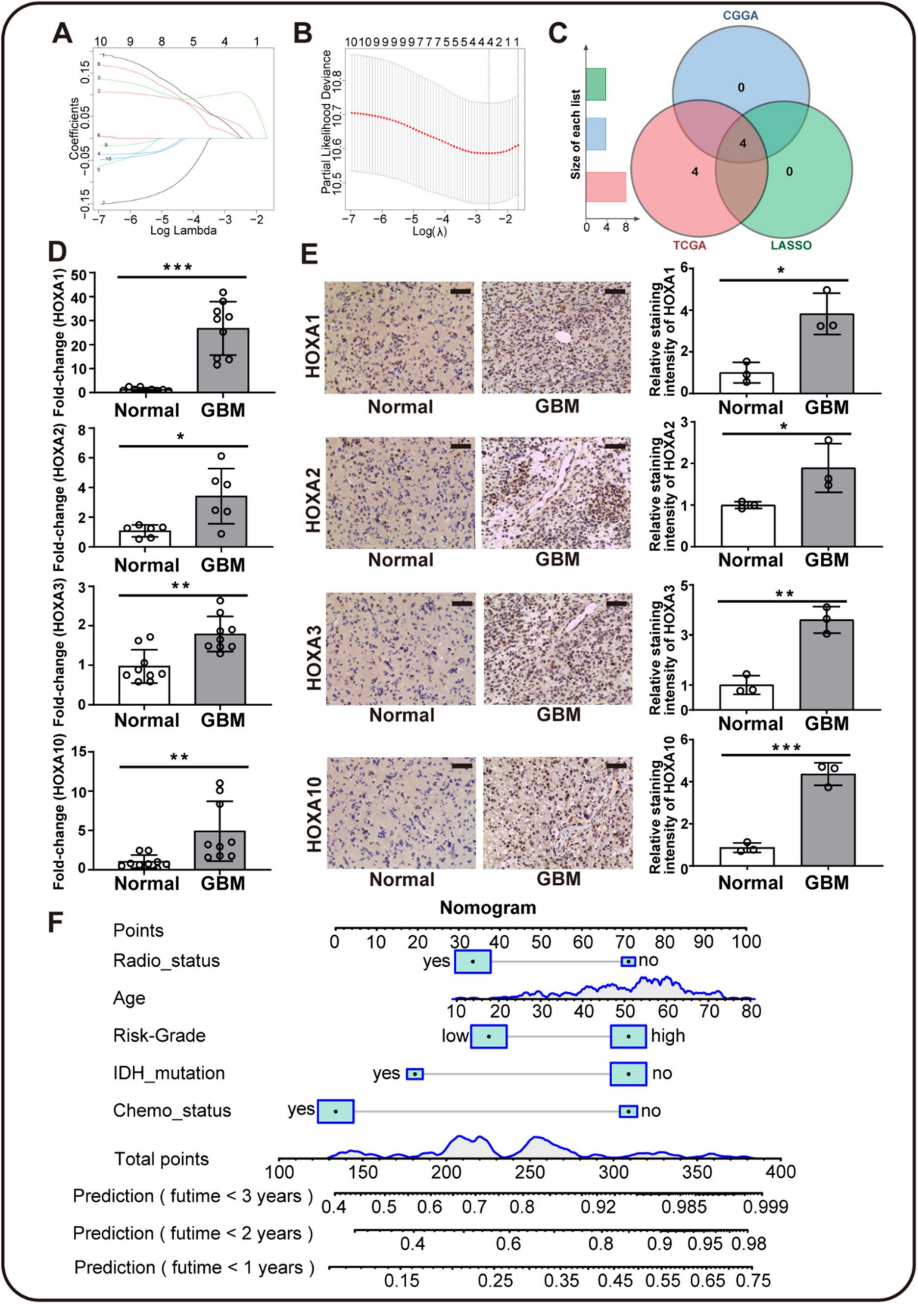
Validation and construction of prognostic HOXAs-based Nomogram model in CGGA and TCGA**.** **A** The least absolute shrinkage and selection operator (LASSO) coefficient profiles of the HOXAs in the CGGA cohort. **B** Selection of the optimal parameter (lambda.1se = 0.07412355) in the LASSO regression model, including HOXA1/2/3/10. **C** Independent prognostic HOXAs, that screened out from CGGA and TCGA were intersected with the optimal parameter from the LASSO regression. **D** Relative HOXA1/2/3/10 mRNA expression in GBM and normal tissues as determined by qRT-PCR. **E** Representative immunochemistry stainings of HOXA1, HOXA2, HOXA3, and HOXA10 in clinical GBM tissues and normal brain tissues. The black scale bar represented 50 μm. Relative staining intensities of HOXA1, HOXA2, HOXA3, and HOXA10 were calculated. (*P < 0.05, **P < 0.01, ***P < 0.001). **F** The nomogram plot was built based on the HOXAs risk-grade, age, radiotherapy, chemotherapy, and IDH mutation. The boxes and curves represented the distribution of the cohort

To further construct a HOXAs-based prognostic prediction model for GBM patients, we performed the regression analysis using the LASSO method from "glmnet" R package. All HOXA members from CGGA database were involved in the regression. The λ value was set at 0.07412355, and four significant HOXA genes were obtained, including HOXA1/2/3/10 (Figs. 7A, B). The results obtained from LASSO and previous multivariate Cox survival analyses of CGGA&TCGA databases were intersected to identify the final optimal HOXA genes (Fig. 7C). Then, qRT-PCR and IHC staining were performed to validate the expression of these HOXA members in GBM and normal tissues. HOXA1, HOXA2, HOXA3, and HOXA10 had high expression in GBM tumor tissues compared to normal tissues (Fig. 7D, E). The results accorded with the expression patterns in multi-databases and supported their potential clinical values. 216 patients from CGGA database were set as the training cohort to construct the Nomogram survival prediction model. The Risk-Grade was calculated based on the expression of HOXAs. The score of Risk-Grade = 0.0890*HOXA1 + 0.0698*HOXA2 + 0.0911*HOXA3 + 0.0436*HOXA10. Then, the median value of the Risk-Grade score was set as the cut-off, which made all samples divided into low and high groups. The Schoenfeld residual test showed that the clinical features and the Risk-Grade satisfied the proportional hazards assumption with *P*-value > 0.05, as shown in Supplementary Fig. 2. The Risk-Grade of HOXAs and clinical features (including age, IDH mutation, radio- and chemotherapy status) had been assigned specific risk scores in the nomogram plot (Fig. 7F). Based on the score points, the model would derive probabilities of 1-, 2-, and 3-year survival times for the patients to further guide the clinical treatment.

### Performance evaluation of Nomogram model based on HOXAs in GBM

**Fig. 8:**
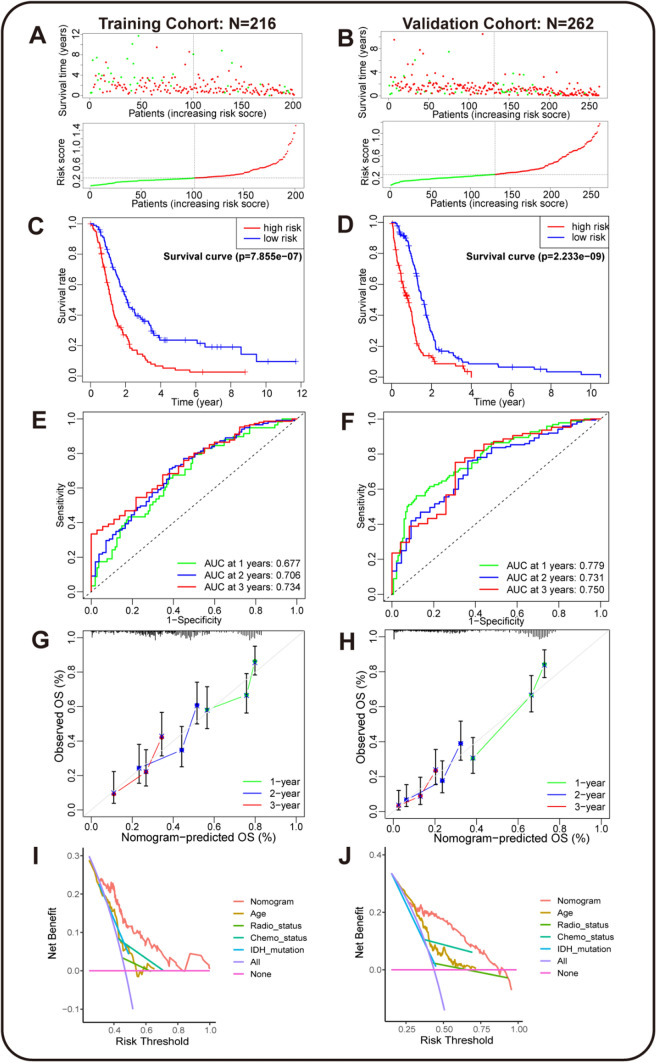
Performance evaluation of HOXAs-based Nomogram model in training (CGGA) and validation (TCGA) sets The distribution of survival time and risk scores in training (**A**) and validation (**B**) cohorts, which was divided by the median risk score of HOXA1/2/3/10. The Kaplan–Meier analyses of the nomogram model in training (**C**) and validation (**D**) cohorts, where the high-risk group was in the red line, and the low-risk was in the blue. **E**, **F** The time-dependent Area Under Curve (AUC) of the nomogram model on 1-, 2-, and 3-year survival time prediction (green, blue, red lines) in training and validation cohorts. **G**, **H** The calibration plots of predicting 1-, 2-, and 3-year (green, blue, red) survival time in the training and validation cohorts. The actual observations were shown in the 45° gray line. **I**, **J** The Decision Curve Analysis (DCA) showed the net benefits of the nomogram model, age, radiotherapy, chemotherapy, and IDH mutation (red, yellow, green, cyan, and blue lines) along with increasing risk threshold. All-treat and no-treat strategies are presented in purple and pink lines

To better utilize the HOXAs-based nomogram model in clinics, we assessed the prediction performance in training and validation cohorts from TCGA and CGGA. The distribution of survival time and risk scores in the training cohort CGGA (216 GBM patients) and validation cohort TCGA (262 GBM patients) were exhibited in Fig. 8A, B. The Kaplan–Meier analysis in the training cohort showed that the high-risk group was significantly correlated with the shorter survival time of GBM patients (Fig. 8C). The correlation was further validated in TCGA cohort, where the high-risk group also had a shorter survival time (Fig. 8D). These results indicated that the nomogram model yielded good prediction performance in the validation. We also conducted more analyses to evaluate the model’s applicability in clinical practice. The time-dependent Area Under Curve (AUC) indicated that the nomogram model had a considerable value in predicting the survival time of GBM patients in the training and validation cohorts (Fig. 8E, F). The AUCs of the nomogram predicting the 1-, 2-, and 3-year survival time were 0.677, 0.706, 0.734 in the training cohort (CGGA); 0.779, 0.731, 0.750 in the validation cohort (TCGA). The prediction ability of the nomogram model was promising. And the calibration plots of the 1-, 2-, and 3-year survival time showed that the predicted values of the nomogram model were in great agreement with the actual observations (Fig. 8G, H). Furthermore, we also utilized the Decision Curve Analysis (DCA) to elucidate the net benefit when the incidence of the disease changes. The nomogram model offered a higher net benefit than the clinical features or all/no treatment strategies (Fig. 8I, J). All the above results were confirmed in the training and validation cohort and supported that the nomogram model could have good clinical applicability in diagnosing and treating GBM patients. The model provided a quantitative method to predict survival time for GBM patients and help clinicians make better medical decisions and follow-up plans.

## Discussion

HOXA family plays complex roles in embryogenesis to tumorigenesis [4, 44], with the gene locus located on chromosome 7. Chromosome 7 trisomy is considered an early event in gliomagenesis and is a pathognomonic characteristic of GBM IDH-wildtype [15]. Despite this, a comprehensive multi-omics analysis of HOXAs in GBM has not been conducted. The present study aimed to uncover the prognostic and diagnostic values of HOXAs in GBM patients and construct a modified risk prediction model based on HOXAs. First, HOXA genes showed high mutation and mRNA expression in pan-cancers, particularly in GBM, as analyzed on GCSA, Oncomine, and TCGA pan-cancers databases. We then validated significantly different mRNA expression levels of HOXAs in normal and GBM samples from CGGA and TCGA. Genetic alteration and Kaplan–Meier survival analysis of HOXA family revealed their prognostic values. We also explored the relationship between HOXAs and clinical GBM classification, including molecular GBM subtypes and IDH mutation, to aid in the diagnosis of GBM patients. We confirmed the independent prognostic roles of HOXA1, HOXA2, HOXA3, and HOXA10, using double multivariate survival analyses and LASSO regression. These HOXAs were further confirmed in GBM tissues via qRT-PCR and IHC staining. Finally, we constructed a nomogram survival prediction model based on the prognostic HOXAs. The model exhibited promising prediction ability and high net benefit in both training and validation cohorts, providing better strategies for diagnosing and treating GBM patients.

Our study confirmed that HOXA1 is highly expressed at both mRNA and protein levels in GBM, consistent with findings from Li and Xia's studies [12, 13]. LncRNA HOTAIRM1 was found to be correlated with the high expression of HOXA1 [13]. The process of epigenetic regulation was involved, such as histone demethylation and sequestered epigenetic modifiers. A study reported that HOXA1 displayed a prognostic role in a single Kaplan–Meier analysis, which was related to cytoskeleton rearrangement in GBM [21]. Our study also obtained a similar result in the Kaplan–Meier analysis and further conducted the multivariate survival analysis in GBM with other clinical factors. We validated the independent prognostic role of HOXA1 in double databases of GBM and first constructed the modified risk prediction model with HOXA1. These findings suggested that HOXA1 has significant potential clinical values in GBM.

We also confirmed the higher expression of HOXA2 in GBM samples, consistent with the results from studies related to glioma [13, 45]. They found that HOXA2 was significantly increased and associated with lncRNA HOTAIRM1 like HOXA1 in glioma. Unlike HOXA1, HOXA2 contributed to the self-renewal of GBM stem-like cells induced by HOTAIRM1 [13]. Liu's research also validated the prognostic value of HOXA2 in glioma patients using Kaplan–Meier analysis with multivariate analysis in CGGA and a single Kaplan–Meier analysis in TCGA [45]. Our study conducted more comprehensive survival analyses, including Kaplan–Meier analysis and multivariate analysis, to confirm the role of HOXA2 in CGGA and TCGA GBM databases. We further demonstrated that the over-expression of HOXA2 was significantly related to the IDH wild-type of GBM, which was also revealed in studies about glioma [45, 46]. Except for the accumulation of DNA methylation, reduced H3K27me3 of HOXAs was also found in the IDH wild-type glioma patients. Overall, our study confirmed the prognostic value of HOXA2 and provided potential targets for further investigation in the epigenetic regulation of HOXA2.

Similar to HOXA2, HOXA3 also contributed to the self-renewal of GBM stem-like cells [13]. Our study confirmed that HOXA3 expression was increased at both mRNA and protein levels in GBM samples compared to the normal. The result aligns with previous studies [13, 14], which found that HOXA3 had a low expression in normal brains but was significantly overexpressed in both GBM and LGGs. The up-regulation in GBM might be correlated with the methylation of HOXA3 [23]. Moreover, we confirmed that the over-expression of HOXA3 contributed to the less survival time of GBM patients. Multivariate survival analysis in CGGA and TCGA confirmed the independent prognostic value of HOXA3 in GBM. Jiang’s study also identified the prognostic roles of HOXA3 in low-grade glioma [14]. While a limited number of studies have investigated the prognostic role of HOXA3 in GBM, our study is the first to demonstrate the independent prognostic role of HOXA3 in GBM comprehensively. Furthermore, antisense RNA of HOXA3 and HOXA11 have also shown prognostic values in glioma [28, 29]. However, these studies were conducted in a single database without external validation from other cohorts. Although antisense RNAs of HOXAs [25–27] have been found to indirectly promote glioma progression, the independent prognostic roles of these non-coding RNAs require further investigation.

HOXA10 was over-expressed in GBM as an independent prognostic member. The over-expression pattern of HOXA10 in GBM was also validated in previous studies [17, 18]. They explored the related regulators of HOXA10, such as the V-ATPase pump and the Trithorax protein mixed lineage leukemia. Furthermore, only one study investigated the prognostic value of HOXA10 in GBM [22]. Interestingly, they found that HOXA10 had a prognostic value in Kaplan–Meier analysis from a single database (236 samples). But further multivariate Cox survival analysis excluded the independent prognostic value of HOXA10. Meanwhile, another research about high-grade pediatric glioma strengthened the prognostic value of HOXA10 via Kaplan–Meier analysis in 78 samples. In our present study, we utilized multiple databases (TCGA and CGGA) that included 478 GBM patients to conduct multivariate survival analyses. The independent prognostic role of HOXA10 in our research would be more convincing. These findings could help us underline the clinical value of HOXAs in GBM.

Besides, IDH mutation [42] and molecular GBM subtypes [43] were also significantly correlated with HOXA family. These classifications were essential clinical markers that guided the diagnosis and treatment of GBM patients. We conducted further correlation analysis and discovered a significant relationship between the HOXA family and IDH mutation and molecular GBM subtypes. The high expression of HOXA1, HOXA2, and HOXA3 was correlated with IDH wild-type. Besides, HOXA1 exhibited significantly different expressions between Proneural and Mesenchymal/Neural GBM subtypes, while HOXA2 and HOXA10 differed in Classical and Proneural subtypes. These findings further reinforce the clinical significance of HOXA family in GBM.

Age, radiotherapy, chemotherapy, and IDH mutation are important clinical elements that guide the diagnosis and treatment of GBM patients. But their net benefits based on these factors were only moderate in CGGA and TCGA groups, as shown in DCA. Previous reviews of nomogram models in GBM also suggested that clinical indicators combined with gene signatures had a better predictive ability [47]. Building upon these findings, we constructed a nomogram survival prediction model based on novel independent prognostic HOXA genes (HOXA1, HOXA2, HOXA3, HOXA10). The model's Kaplan–Meier analyses and AUC values were promising in the training cohort and higher in the validation cohort. Compared to previous prediction models [47, 48], the performance of our model was acceptable, with the AUC level in the validation group of > 0.75 at 1-year/3-year, which was also similar to the deep-learning and machine-learning models (C-index = 0.68–0.80) [48]. The prediction performance and net benefits of the model supported its clinical applicability. As a novel quantitative method, our model could help clinicians make better medical decisions and follow-up plans for GBM patients.

The abnormal expressions of HOXAs in GBM, especially these prognostic members, could be related to the complex regulation processes in GBM. In addition to the dysregulation of normal functions of HOXAs, the tumor progression would promote copy-number variations and epigenetic alteration. Chromosome 7 trisomy and chromosome 10 monosomy were common in IDH-wildtype GBM patients. Notably, the HOXA locus is also located on chromosome 7, which is related to the expression of HOXA1 [46]. Gallo’s study [17] and our research both found that HOXA10 showed no significant changes in IDH-wildtype GBM. However, HOXA2 in our study was significantly increased in IDH-wildtype GBM, which differed from the previous study [46]. Due to the special locus site of HOXA, the copy-number variations and their roles in HOXA family still needed more investigation. In normal development, the expressions of HOXAs were regulated by polycomb group proteins and H3K27me3. In cancer development, these genes could promote aberrant DNA methylation [49]. High methylation of HOXA3 and low level of HOXA10 were found in GBM [23]. Our findings of prognostic HOXAs suggested that the regulation of HOXAs might also correlate with the prognosis of GBM patients. Although we spared no effort to analyze HOXAs in multiple databases comprehensively, this study was still far from flawless. First, we conducted experimental validation only on prognostic HOXA genes, such as the qRT-PCR and IHC staining of HOXA1-3 and HOXA10. The rest of HOXAs was lack of further validation. Besides, this study did not investigate tumorgenesis-related pathways and in-depth mechanisms. These novel directions will be more focused on in our forthcoming research.

In summary, our study found that HOXA family was highly mutated and expressed in GBM. The genetic alteration and mRNA expression of HOXAs were prognostic indicators. HOXA1/2/3/10 were independent prognostic factors that were validated by multivariate survival analyses, lasso regression, qRT-PCR, and IHC staining. We constructed The HOXAs-based nomogram survival prediction model that exhibited significant clinical utility in GBM. this study provided comprehensive insights into the clinical significance of HOXA family in GBM and suggested a promising new strategy for diagnosing and treating GBM patients.

## Supplementary Information

Additional file1

Additional file2

Additional file3

Additional file4

## Data Availability

The datasets analyzed in this study are available in the TCGA (https://portal.gdc.cancer.gov/) and CGGA (http://www.cgga.org.cn/) repositories.

## Abbreviations

HOXA: Homeobox A
GBM: Glioblastoma
OS: Overall survival time
CGGA: The Chinese Glioma Genome Atlas
TCGA: The Cancer Genome Atlas
LASSO: The least absolute shrinkage and selection operator
qRT-PCR: Quantitative real-time PCR
IRB: Institutional Review Board
IHC: Immunohistochemical
GSCA: Gene Set Cancer Analysis
DAB: Diaminobenzidine
AUC: Area Under Curve
DCA: Decision Curve Analysis

## Code availability

The R packages used in this study are listed in Supplementary Table S3.
