# Supplementary material for: Development and validation of a predictive model in diagnosis and prognosis of primary glioblastoma patients based on Homeobox A family
Source: Discov Oncol. 2023 Jun 23;14:108. doi: 10.1007/s12672-023-00726-y (PMC10290013; doi:10.1007/s12672-023-00726-y)
Supplement: Supplementary file 1 — Additional file1 [file 12672_2023_726_MOESM1_ESM.docx]

**Supplemental Information**

Development and validation of a predictive model in diagnosis and prognosis of primary glioblastoma patients based on Homeobox A family

Gui-Qiang Yuan^1, 2^, Guo-Guo Zhang^1^, Qian-Qian Nie^1^, Zong-Qing Zheng^1*^, Zhong Wang^1^**^*^**

^1^Department of Neurosurgery & Brain and Nerve Research Laboratory, The First Affiliated Hospital of Soochow University, Suzhou, Jiangsu Province, China.

^2^Beijing Neurosurgical Institute & Department of Neurosurgery, Beijing Tiantan Hospital Affiliated to Capital Medical University, Capital Medical University, Beijing, China

^*^Correspondence:

Corresponding Author: Zhong Wang, Zong-Qing Zheng, Department of Neurosurgery, The First Affiliated Hospital of Soochow University, 188 Shizi Street, Suzhou 215006, China. Tel: +86-18100684632; Fax: +86-051267787170. Email: wangzhong_8761@163.com, zongqing22@u.nus.edu.

**Content:**

1. Supplemental Figure (1-2)
2. Supplemental Figures legend (1-2)
3. Supplemental Tables (1-6)
4. **Supplemental Figure:**

**Supplemental Figure 1.**


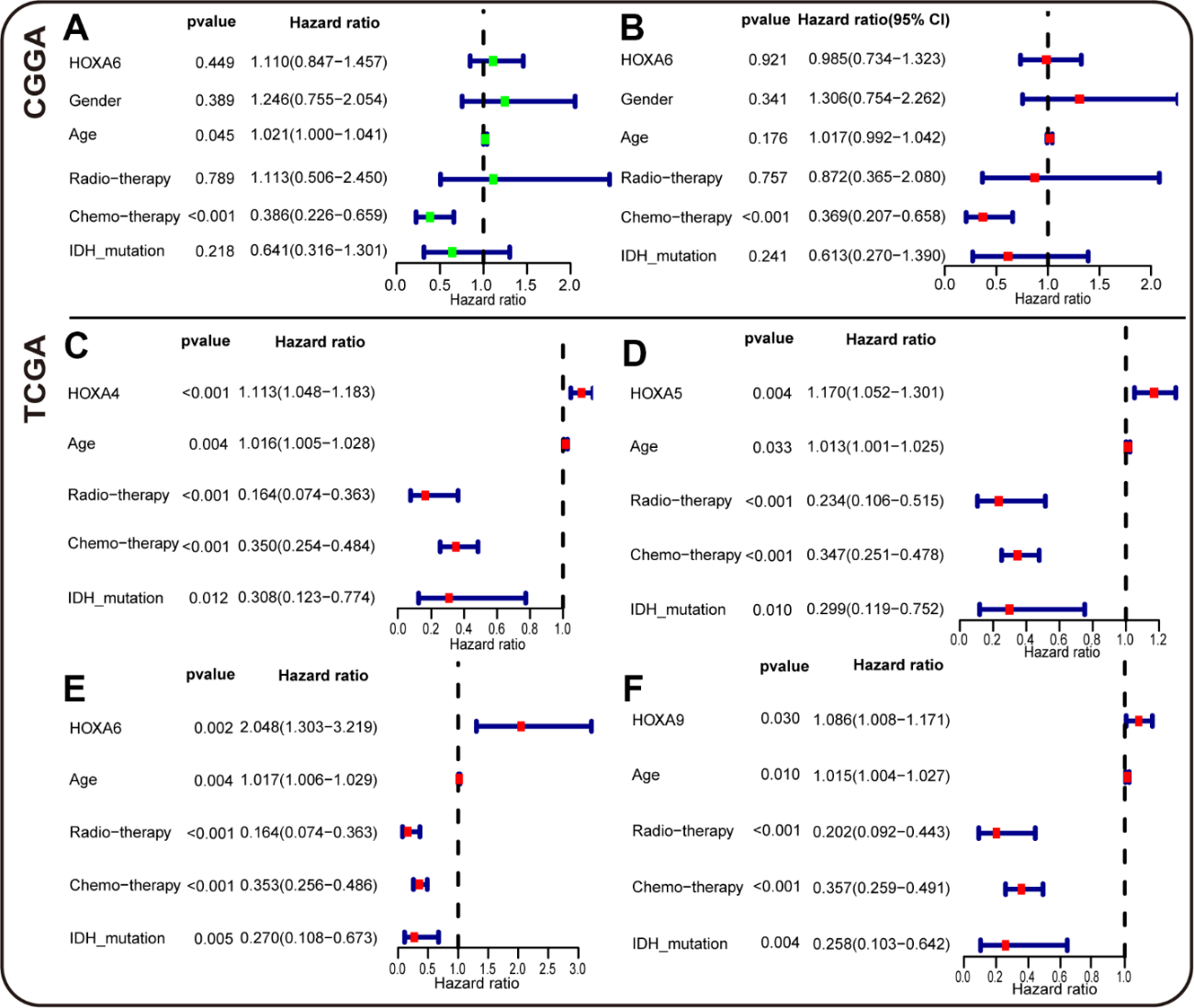


**Supplemental**
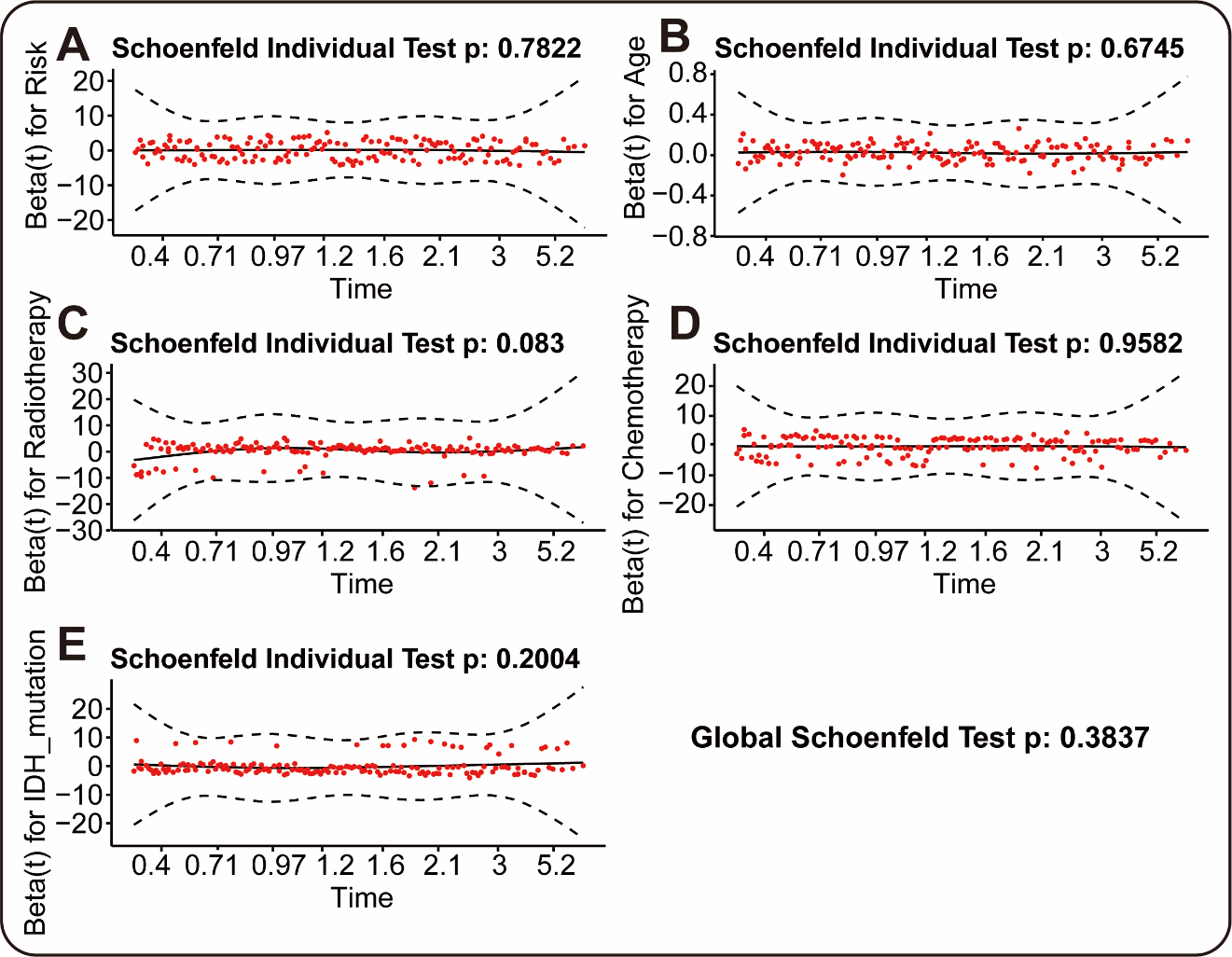
**Figure 2.**

1. **Supplemental Figures legend:**

**Supplemental Figure 1.** **Prognostic values of rest HOXAs in CGGA and TCGA databases**. **(A)** The univariate survival analysis of HOXAs and clinical factors in the "mRNAseq_325" CGGA database by "survival", "survminer" R packages. HOXA6, gender, age, radiotherapy, chemotherapy, and IDH mutation were included. (The dots in the plots were green.) **(B)** Further multivariate survival analysis of HOXA6 and clinical factors (gender, age, radiotherapy, chemotherapy, IDH mutation) in CGGA. (The dots in the plots were red.) **(C-F)** Multivariate survival analysis of HOXA4, HOXA5, HOXA6, HOXA9, and clinical factors (gender, age, radiotherapy, chemotherapy, IDH mutation) in TCGA database. (The dots in the plots were red.)

**Supplemental Figure 2. Visualization of Schoenfeld residual test of** **proportional hazards (PH) assumption for clinical characteristics.** The risk scores **(A)**, and clinical features, including age **(B)**, radiotherapy **(C)**, chemotherapy **(D),** and IDH mutation **(E)**, satisfied the PH assumption with P-value >0.05. The P- value of global Schoenfeld test was 0.3837.

1. **Supplemental Tables:**

**Supplemental Table 1. Basic characteristics of 216 GBM patients in CGGA.**

| **Variables** | |
| --- | --- |
| Gender (Male/Female) | 130/86 |
| Age (Years, Mean ± SD) | 50.95±12.86 |
| Survival Time（Days, Median） | 668.8(0-3802) |
| Censor | N=216 |
| Radiotherapy | N=209 |
| Chemotherapy | N=208 |
| IDH Mutation | N=209 |
| Expression of HOXA1, HOXA2, HOXA3, HOXA4, HOXA5, HOXA7, HOXA9, HOXA10, HOXA11, HOXA13 | N=216 |
| Expression of HOXA6, | N=84 |

GBM: Glioblastoma multiforme, SD: standard deviation.

**Supplemental Table 2. Basic characteristics of 262 GBM patients in TCGA.**

| **Variables** | |
| --- | --- |
| Gender (Male/Female) | 170/92 |
| Age (Years, Mean ± SD) | 60.05±13.45 |
| Survival Time（Months, Median） | 16.16(0.200-127.6) |
| Censor | N=262 |
| IDH Mutation | N=262 |
| Expression of HOXA1, HOXA2, HOXA3, HOXA4, HOXA5, HOXA6, HOXA7, HOXA9, HOXA10, HOXA11, HOXA13 | N=262 |

GBM Glioblastoma multiforme, SD: standard deviation.

**Supplemental Tables 3. R** **programming language used R packages.**

| **Analysis** | **R packages** |
| --- | --- |
| Heatmap of mRNA expression in pan-cancers/GBM | "pheatmap" |
| Boxplot of mRNA expression in pan-cancers/GBM | "ggpubr" |
| Kaplan-Meier | "survival", "survminer" |
| Cox analysis and multi-Cox | "survival", "survminer" |
| ROC | "timeROC" |
| Features of the risk model | "limma", "pheatmap" |
| Lasso regression | “glmnet” |
| IDH mutation | "beeswarm" |
| Clinical elements | “limma”, "ggplot2", "reshape2" |
| Tests of proportional hazards assumption | "survminer" |

R programming language version 4.0.5.

**Supplementary Table 4. Datasheet of GBM patients from CGGA.**

Attached Table S4.txt file.

**Supplementary Table 5. Datasheet of HOXA6 in GBM patients from CGGA.**

Attached Table S5.txt file.

**Supplementary Table 6. Datasheet of GBM patients from TCGA.**

Attached Table S6.txt file.
